# Novel use of arm ergometry cardiopulmonary exercise testing for non-elective patients with chronic limb-threatening ischaemia: a feasibility trial

**DOI:** 10.1136/bmjopen-2026-118841

**Published:** 2026-06-15

**Authors:** Stacie Hodge, Adam Haque, Tanviha Quraishi-Akhtar, Angella Bryan, Steven Rogers, Frank Lee Bowling, Jonathan Ghosh

**Affiliations:** 1 Division of Cardiovascular Sciences, The University of Manchester, Manchester, UK; 2 Manchester Academic Vascular Research and Innovation Centre (MAVRIC), Manchester University NHS Foundation Trust, Manchester, UK; 3 Department of Anaesthesia, Manchester University NHS Foundation Trust, Manchester, UK; 4 Manchester Academic Health Science Centre, Manchester, UK; 5 School of Medical Sciences, The University of Manchester, Manchester, UK

**Keywords:** Vascular surgery, Multimorbidity, Cardiovascular Disease

## Abstract

**Objectives:**

To evaluate the feasibility, safety and acceptability of arm crank ergometry cardiopulmonary exercise testing (CPETarm) in patients with chronic limb threatening ischaemia (CLTI).

**Design:**

Prospective feasibility single-arm cohort study.

**Setting:**

A tertiary vascular surgery referral centre in Greater Manchester, UK.

**Participants:**

Adult inpatients admitted with CLTI and scheduled for non-elective vascular intervention.

**Interventions:**

Participants underwent bedside CPETarm using an incremental ramp protocol. Cardiopulmonary parameters measured included peak oxygen uptake, anaerobic threshold (AT), ventilatory equivalent for carbon dioxide at AT, peak work rate, oxygen pulse, maximum heart rate and respiratory exchange ratio.

**Primary and secondary outcome measures:**

Primary outcomes were feasibility domains including eligibility, recruitment, test completion, safety, practicality, implementation and patient acceptability. Secondary outcomes included the ability to obtain clinically relevant CPET variables for perioperative risk stratification.

**Results:**

60 patients underwent CPETarm. 74% of CLTI inpatients met eligibility criteria and 71% of eligible patients consented to testing. CPETarm was completed to volitional exhaustion by 95% of participants, with anaerobic threshold identified in 68%. No major adverse events occurred during testing or within 24 hours post-test. 90% of CPETarm assessments were completed within 48 hours of the decision to proceed with intervention, without delaying surgery. The procedure was well tolerated and acceptable to patients.

**Conclusions:**

CPETarm is a feasible, safe and acceptable method for preoperative assessment in patients with CLTI who are unsuitable for conventional lower-limb CPET. Further research is required to establish modality-specific thresholds, evaluate prognostic value for postoperative outcomes and evaluate integration into perioperative care pathways.

**Trial registration number:**

NCT06404229.

**STRENGTHS AND LIMITATIONS OF THIS STUDY**

- Focuses on a high-risk surgical population who are often unsuitable for standard preoperative testing.
- Development of a novel population specific cardiopulmonary exercise testing protocol, addresses limitations of existing protocols designed for healthy individuals.
- A mixed methods feasibility assessment with strong patient involvement enhances relevance, acceptability and future scalability.
- The results may reflect specific local expertise and equipment, potentially limiting generalisability to other clinical settings.

## Introduction

 Chronic limb threatening ischaemia (CLTI), characterised by ischaemic rest pain and/or tissue loss, represents the most advanced stage of peripheral arterial disease. ^1 2^ It is associated with significant morbidity, including chronic pain, major amputation and increased mortality, often related to the complex comorbid profiles common in this patient population. Prompt revascularisation is crucial for limb salvage and survival^3^; however, surgical intervention carries a high risk. 30-day postoperative mortality following infrainguinal surgical revascularisation has been reported to range from 3%^4 5^ to as high as 15%, among high-risk groups.^5^ This risk profile is further exacerbated as nearly 50% of patients present as an emergency,^2^ making preoperative assessment challenging.

Cardiopulmonary exercise testing (CPET) provides a comprehensive assessment of the cardiorespiratory system under stress of incremental or maximal exertion. It is considered the gold standard tool for evaluating cardiorespiratory fitness and is performed increasingly for preoperative assessment. Variables such as peak oxygen uptake (VO_2_), ventilatory equivalent of carbon dioxide (VE/VCO_2_) and anaerobic threshold (AT) are associated with both short- and long-term outcomes.^6^,^9^ Traditionally, CPET is conducted using cycle ergometry (CPETleg) or treadmill testing.^10 11^ However, these modalities pose significant challenges for patients with lower limb dysfunction, such as those with CLTI. Arm-crank ergometry CPET (CPETarm) offers a possible alternative, but this is yet to be tested in a CLTI population.

While CPETarm has been shown to provide reliable data on aerobic capacity in both healthy individuals^12^ and those with cardiovascular disease,^13^ it tends to yield lower AT and peak VO_2_ values when compared with lower limb CPET.^14^ Reduced skeletal muscle volume and differing metabolic responses of the upper body lead to an increase in anaerobic metabolism,^14^,^16^ with muscle deoxygenation occurring in the triceps at 50% of peak VO_2_ compared with >80% in the legs.^17^ Notably, the difference in performance between arm and leg modalities is smaller in older adults and in individuals with reduced aerobic capacity.^18^ CPETarm has also demonstrated clinical utility for detecting myocardial ischaemia in patients unable to perform conventional leg exercise.^19 20^

These findings support the potential application of CPETarm for preoperative risk stratification, particularly in patients who are unable to undertake traditional cycle ergometry, such as those with CLTI. This prospective cohort study explores the feasibility of CPETarm in non-elective patients admitted with CLTI and considers safety, practicalities and acceptability.

## Methods

### Design and population

Patients admitted with CLTI and scheduled for non-elective vascular surgical intervention between August 2024 and March 2025 at Manchester University NHS Foundation Trust were eligible for inclusion. The inclusion criteria were: (1) confirmed diagnosis of CLTI, as defined by the Global Vascular Guidelines^1^ and (2) decision made by the patient’s Consultant Vascular Surgeon for open, endovascular or hybrid revascularisation or primary major limb amputation. Exclusion criteria were: (1) active medical conditions deemed an absolute or relative contraindication to CPET as guided by established clinical guidelines;^6^ (2) physical disabilities preventing the patient from undertaking CPET; (3) psychiatric disorder or dementia that impaired the patient’s ability to consent, participate in testing or complete follow-up. The trial was registered with ClinicalTrials.gov (NCT06404229). Patients were recruited consecutively following emergency admission for CLTI, with testing aimed to be performed within 72 hours and prior to surgery. CPET results were withheld from the clinical team unless a clinical adverse event occurred, in which case the findings were disclosed.

### Patient and public involvement

Our patient and public involvement and engagement (PPIE) panel comprised of people living with cardiovascular disease. The panel was involved from the earliest stages of the research and contributed to shaping the study protocol. To improve clarity, accessibility and acceptability panel members also provided input into the design of the participant information sheets, consent form and post-trial evaluation.

### Cardiopulmonary exercise testing

Immediately prior to CPET, participants performed spirometry to derive forced expiratory volume over 1 second and forced vital capacity which were used to assess ventilatory reserve as determined by validated methods^21 22^ and ensure both safety and accuracy of the test.^23^ This approach is standard in our institution’s CPET service.^23^

Participants then underwent CPET using a calibrated electromagnetically braked arm ergometer (Lode Angio, Groningen, Netherlands). Previously described CPETarm ramp protocols in healthy volunteers^24^ were deemed unsuitable for this CLTI population, due to significant functional differences. Instead, a novel protocol was co-developed through collaborative focus testing between the research team and patients. The protocol required participants to perform 3 min of unloaded cycling using the arm ergometer (0 watts (W)) as warm up, before an incremental ramp phase. The incremental ramp protocol (2.5 W/min, 5 W/min or 8 W/min) was determined by handgrip strength measurements, using a hand dynamometer (Marsden MG-4800, Rotherham, UK). The arm ergometer was adjusted to ensure alignment between the ergometer’s crankshaft and the participant’s glenohumeral joint. Seating position maintained slight elbow flexion at the point of maximal reach.

Previously reported data suggests that arm crank speeds between 50 and 80 revolutions per minute (rpm) have no influence on peak VO_2_,^25^ therefore, participants were instructed to select a comfortable cadence within this range and maintain it consistently until they reached volitional exhaustion. Exhaustion was defined as a decrease in cadence of more than 10 rpm for five consecutive seconds or a cadence falling below 45 rpm despite verbal encouragement. Due to a lack of established criterion to demonstrate maximal effort for CPETarm, the following standards were used to suggest sufficient patient effort; a plateau in VO_2_, maximal respiratory exchange ratio (RER) exceeding 1.15^22^ and/or achieving at least 80% of their predicted maximum heart rate.^26^

Pulmonary gas exchange was continuously monitored using a metabolic cart (Vyntus CPX, Vyaire Medical, Mettawa, Illinois, USA) with gas and flow calibration performed prior to each test. Blood pressure measurement was taken before and after exercise. ECG and pulse oximetry (SpO_2_) were continuously monitored throughout. CPET was terminated if any ischaemic ECG changes (horizontal or down sloping ST-segment depression of greater than 1 mm, persisting for over 80 ms) or significant desaturation was detected.

CPET parameters measured included peak VO_2_, AT (as determined by the V-slope method^27^), VE/VCO_2_, peak work (measured in watts), oxygen pulse, maximum heart rate and RER. Results are presented as absolute values. Predicted maximum heart rate was calculated using the equation published by Tanaka *et al*.^28^ For participants on beta-adrenergic blocking agents, the predicted maximum heart rate was estimated using the equation formulated for individuals with coronary artery disease.^29^

### Assessment of feasibility

Feasibility was assessed in accordance with established guidelines^30^ and included assessment across four domains:

1. Safety.
2. Implementation.
3. Practicality.
4. Acceptability for large scale evaluation and demand.

Acceptability and demand were assessed by gathering participants’ satisfaction and perceptions of, and suitability of CPET for future use. This process involved two components which were performed on completion of CPET. First, participants rated their agreement with 12 brief statements using a 5-point Likert scale. Second, qualitative feedback was collected through a semi-structured interview. Interviews were conducted at bedside, each lasting approximately 5–10 min by the lead researcher (SH). Responses were audio-recorded and summarised descriptively. Data were analysed using thematic analysis. An initial coding framework was developed and applied across transcripts, with codes subsequently organised into broader themes. A second researcher independently reviewed a subset of transcripts with discrepancies resolved through discussion. Given the focused scope of the study, the analysis aimed to identify key patterns in participant perspectives rather than achieve formal data saturation.

### Clinical outcomes

Binary secondary outcomes measured at 30 and 90 days included: hospital readmission (defined as inpatient admission to an acute care hospital following a discharge), any major adverse limb event (MALE; defined as untreated loss of patency, reintervention on the index arterial segment or major amputation of the index limb), any major adverse cardiac event (MACE; defined as myocardial infarction, stroke or cardiovascular death) and all-cause mortality.

### Statistical analysis

Baseline anthropometric, spirometry and clinical data were compared between sexes using independent samples t-tests. Data were presented as pooled mean value±SD. Additionally, effect sizes were calculated according to established thresholds^31^ to determine the magnitude of differences between groups: trivial (<0.2), small (0.2–0.5), medium (0.5–0.8) and large (≥ 0.8). All analyses were undertaken using GraphPad Prism and a p<0.05 was considered significant.

Our research team, internal peer review and PPIE group agreed a sample size of 60 was sufficient to assess feasibility. This sample size also meets the minimum of 30 participants to assess intervention feasibility recommended in published guidelines.^32^

## Results

### Participants

60 patients with CLTI were recruited for the study, of whom 32% were female (n=19). Baseline demographics are listed in table 1.

**Table 1:** Baseline anthropometric, clinical and pulmonary data, alongside differences between sexes in 60 patients with CLTI

Variable	All (n=60)	Male (n=41)	Female (n=19)	P value	ES
Age (years)	65 ±11.6	67 ± 9.9	63 ±14.7	0.28	0.31
Height (cm)*	168 ± 9.4	173 ± 7.8	160 ±5.4	**<0.001**	**1.96**
Mass (kg)	76.7 ±22.2	81.7 ±20.5	65.9 ± 22.3	**0.013**	0.74
BMI (kg/m^2^)	26.7 ± 7.1	27.3 ± 6.8	25.4 ± 7.6	**<0.001**	0.27
Haemoglobin (g/L)	120 ± 19.5	122 ±18.5	117 ±21.8	0.347	0.27
Hand grip strength (kg)	26.3 ±10.5	30.8 ± 9.31	16.7 ±5.4	**<0.001**	**1.85**
FEV_1_ (L)	2.26 ± 0.76	2.50 ±0.70	1.75 ±0.65	**<0.001**	**1.11**
FEV_1_ (% predicted)	82 ± 18.8	83 ± 19.8	78 ± 16.3	**<0.001**	0.28
FVC (L)	3.28 ± 1.1	3.66 ±0.95	2.45 ±0.88	**<0.001**	**1.31**
FVC (% predicted)	94 ± 21.5	94 ± 21.3	93 ±22.6	**<0.001**	0.08
FEV_1_/FVC	0.70 ± 0.12	0.68 ±0.12	0.71 ±0.10	0.322	0.26

All continuous variables reported as mean±SD. Significant differences (p<0.05) and large effect sizes (≥0.8) between groups highlighted in bold.

* For patients with a history of major limb amputation on the contralateral side, ulnar length was measured to derive an estimation of height.

BMI, body mass index; CPET, cardiopulmonary exercise test; ES, effect size; FEV1, forced expiratory volume in 1 s; FVC, forced vital capacity.

Based on grip strength, 40% of patients (n=24) met the criteria for sarcopenia.^33^ Anaemia was present in 68% of men (n=28) compared with 53% of females (n=10). 28% of patients (n=17) had a Clinical Frailty Score of 5 or more. Multimorbidity was observed in 72% of patients (n=43) 27% (n=16) were people who smoke currently and 48% (n=29) people who have quit smoking.

The Global Initiative for Chronic Obstructive Lung Disease criteria^34^ were used to determine patterns of respiratory disease. A mild obstructive pattern was observed in 18% of patients (n=11), 20% (n=12) demonstrated a moderate obstruction and 5% (n=3) a severe obstructive pattern. In individuals with identified obstructive abnormalities (n=26), only 38% (n=10) had a prior diagnosis of chronic obstructive pulmonary disease. Notably, 75% (n=9) with moderate obstruction and 33% (n=1) with severe obstruction were previously undiagnosed.

### Safety

No major adverse events occurred during or within 24 hours of CPET. Two tests were terminated early for safety reasons as the ECG response could not be properly monitored due to a poor trace. One participant’s ECG reading during CPET revealed ventricular ectopics. Referral for 24-hour ECG monitoring and echocardiogram were subsequently made. Post exercise, one participant reported new onset chest pain. Cardiology assessment including review of stress ECG and investigations for acute coronary syndrome led to a diagnosis of musculoskeletal chest pain. No post-exercise systolic blood pressures ≥200 mm Hg, or diastolic pressures ≥110 mm Hg were recorded, and no other complications reported.

### Implementation

115 patients were screened for inclusion with 26% (n=30) excluded, 50% (n=15) of which were due to active medical conditions deemed a contraindication for undertaking CPET (undergoing evaluation for coronary artery disease; n=9, active arrhythmia; n=3, requiring up-titration of diuretics; n=1 and dependence on oxygen therapy; n=2). 27% (n=8) were excluded due to a physical disability precluding CPET, including demyelinating polyneuropathy (n=1), shoulder pain (n=4), hemiplegia from cerebrovascular event (n=2) and inability to sit upright (n=1). A further 23% (n=7) did not have capacity to consent to the study.

**Figure 1:**
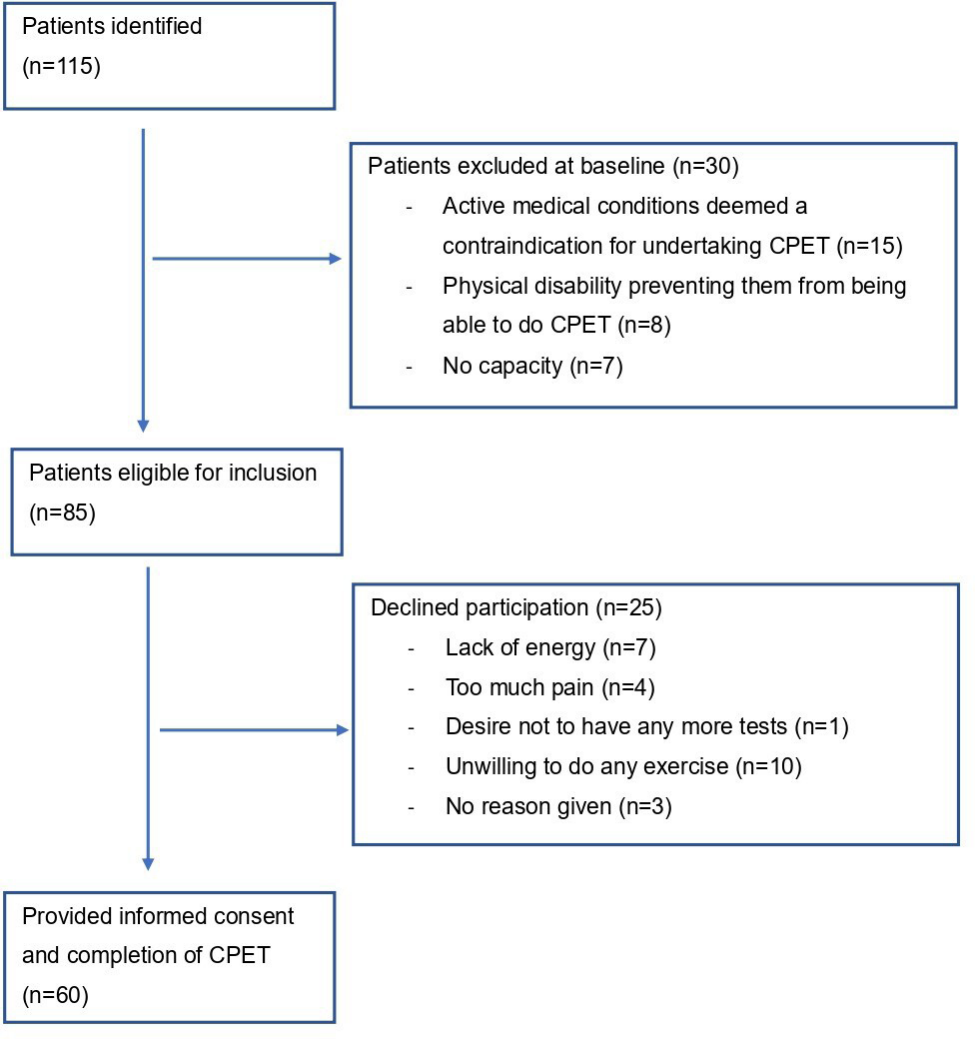
Flow chart detailing inclusion, exclusion and withdrawal of participants during study period. CPET, cardiopulmonary exercise testing.

Of the 85 eligible, 71% (n=60) provided informed consent with 29% (n=25) declining participation (citing; lack of energy; n=7, too much pain; n=4, desire not to have any more tests; n=1, unwillingness to exercise; n=10 and no reason given; n=3), see figure 1.

The majority of patients (n=57, 95%) were able to complete CPET to volitional exhaustion. Of the 5% (n=3) who failed to reach exhaustion, the reasons for clinical cessation included; poor ECG trace leading to a precautionary termination (n=2) and excessive musculoskeletal pain (n=1).

### Practicality

During exercise, all participants were able to maintain their cadence as instructed; the majority (82%) self-selected a cadence of 50–60 rpm, while 3% selected a cadence >70 rpm. Age-predicted maximal heart rate >80% was achieved by 40% of patients (n=17) not established on beta blocker therapy, and by 24% (n=4) on beta blockers. RER >1.15 was achieved by 45% of participants (n=27). AT was achieved in 68% of patients (n=41). Reason for stopping the test was arm fatigue or pain in 65% (n=39), general fatigue/tiredness in 13% (n=8) and breathlessness in 22% (n=13).

The majority (93%) of CPETs were completed within 72 hours of the decision to initiate treatment, 54 tests (90%) within 48 hours, with a median time of 21 hours and 45 min (IQR 3 hours 58 min to 30 hours 39 min). The median total test duration was 10 min and 39 s (IQR 7 min 42 s to 13 min 12 s).

### Acceptability and demand

All participants (n=60) completed the post-trial evaluation. Responses for each question are provided in table 2.

**Table 2:** Quantitative responses to questions pertaining to exercise testing and the role of CPETarm for patients with CLTI

Question	Mean±SD	Range
1. ‘When I first heard about this trial, I was keen to be involved’	4.50±0.72	2–5
2. ‘From the information provided, I understood exactly what the test was for’	4.77±0.50	3–5
3. ‘Understanding more about my health and the risk of undergoing surgery is important to me’	4.90±0.30	4–5
4. ‘The idea of using exercise testing to assess risk prior to surgery appeals to me’	4.78±0.62	2–5
5. ‘The time I waited for the test was reasonable’	4.93±0.25	2–5
6. ‘I did not feel that testing delayed any of my other treatment’	4.98±0.13	4–5
7. ‘The instructions were easy to follow’	4.98±0.13	4–5
8. ‘The exercise test was too challenging for me’	1.38±0.55	1–4
9. ‘The test was of an appropriate duration’	4.83±0.62	1–5
10. ‘My understanding of what the test would involve turned out to be realistic’	4.88±0.37	3–5
11. ‘Overall involvement with the trial has been a positive experience’	4.92±0.28	4–5
12. ‘Based on my experience in this trial, I think cardiopulmonary exercise is acceptable for people with the same condition as mine’	4.87±0.43	3–5

CLTI, chronic limb threatening ischaemia; CPETarm, arm-crank ergometry cardiopulmonary exercise testing.

Feedback clustered around the following themes: (1) desire for a better understanding of current health, (2) improving readiness for surgery and identifying potential risks, (3) value of clear physiological data and being able to ‘see the results’, (4) reassurance and increased confidence prior to surgery. Examples of answers provided the semi-structured interview are provided in table 3.

**Table 3:** Selected responses to questions pertaining to exercise testing and the role of CPETarm for patients with CLTI

Theme	Direct participant quotes
A desire for a better understanding of current health	“Knowing what I can do and what I thought I could do” “Just to see how good I would do. I am keen on keeping myself alive” “I want to see what’s going on, on the inside, so I can change my lifestyle” “I wanted a better understanding of my health” “Showing you whether the lifestyle choices you made have been the right ones for you”
Improving readiness for surgery and identifying potential risks	“I wanted to test myself, seeing as I am going for surgery” “It looks at your fitness prior to an operation” “You may pick up something that the person needs fixing before they can have an operation” “I wanted to understand more about the effect of surgery on my body”
Value of clear physiological data and being able to ‘see the results’	“It’s all really interesting, seeing how the heart and lungs work together” “The readouts of what my heart and lungs are doing” “It’s good to be able to see the results so clearly” “The informative aspect is key” “I can see my heart is doing what it should be doing”
Reassurance and increased confidence prior to surgery	“It gave me confidence” “It’s reassuring more than anything” “I feel like I’ve had a full MOT”

CLTI, chronic limb threatening ischaemia; CPETarm, arm-crank ergometry cardiopulmonary exercise testing.

Broadly, CPET was viewed positively.

It’s a good idea. It looks at your fitness prior to an operation. I don’t want to go in and not come out.

I think it’s an easy way to assess people prior to surgery. Point out any faults that might delay surgery.

It’s the informative aspect which is key.

10% of participants however described some difficulties:

Because I’ve not exercised for a while, when my muscles started hurting, I decided to stop whereas with practice I might be able to do more.

I didn’t like the mask, it felt claustrophobic.

The design of the arm ergometer, it should be more lightweight and accessible.

For patients who had previously done previous exercise tests (n=12), CPET was preferred by 58% (n=7).

I’ve had previous tests for breathing and my heart but never had a test which looks at them both.

Did a bike test 17 years ago, I was a lot fitter then. I couldn’t do that now and would prefer to do this.

I have done the step test “up and go”. This is better because you are sat down.

### Physiological values

The median peak VO_2_ for this cohort was 11.4 mL/kg^−1^/min^−1^ (IQR 9.3–13.6) with a significant difference observed between sexes (p<0.001) (table 4).

**Table 4:** Key CPET parameters measured, alongside differences between sexes in 60 patients with CLTI

Variable	All (n=60)	Male (n=41)	Female (n=19)	P value	ES
Watts	31 ±17.2	32 ± 18.0	27 ±15.3	0.229	0.32
Cadence (rpm)	56 ± 6.2	57 ± 7.1	54 ± 3.0	**0.012**	0.62
VO_2peak_ (mL/kg/min)	11.4 ± 3.0	11.1 ±2.9	12.1 ± 3.3	**<0.001**	0.30
Maximum heart rate (bpm)	108± 22.5	105 ±21.3	115 ±24.5	0.155	0.42
Maximum heart rate (% predicted)	73 ±14.5	72 ± 14.5	77 ± 15.2	0.265	0.32
VO_2_/HR	8.4± 3.5	9.1 ± 3.9	6.7 ±1.8	**0.002**	0.79
VO_2_/HR (% predicted)	74 ± 22.9	70 ± 25.3	81 ± 15.1	0.056	0.49

All continuous variables reported as mean±SD. Significant differences (p<0.05) and large effect sizes (≥0.8) between groups highlighted in bold.

bpm, beats per minute; CPET, cardiopulmonary exercise test; ES, effect size; HR, heart rate; rpm, revolutions per minute; V0_2peak_, peak oxygen uptake.

Among participants with an identifiable AT (n=41), the median VO₂ at AT was 8.9 mL/kg^−1^/min^−1^ (IQR 7.9–9.9), corresponding to 72% of the achieved peak VO_2_. No significant differences were observed between sexes for values at AT (table 5). The median VE/VCO_2_ at AT was 34.3 (IQR 30.4–36.1) and median peak work rate was 31 W (IQR 17–45).

**Table 5:** Key CPET parameters measured, alongside differences between sexes, in patients achieving anaerobic threshold

Variable	All (n=41)	Male (n=29)	Female (n=12)	P value	ES
VO_2_ at AT	9.1 ± 2.0	8.9 ± 1.5	9.6 ± 3.0	0.49	0.33
Watts at AT	25 ± 9.9	25 ± 10.0	24 ± 10.2	0.77	0.10
VE/VCO_2_ at AT	34.7 ± 5.6	35.5 ± 6.2	32.8 ± 3.6	0.92	0.53

All continuous variables reported as mean±SD. Significant differences (p<0.05) and large effect sizes (≥0.8) between groups highlighted in bold.

AT, anaerobic threshold; CPET, cardiopulmonary exercise test; ES, effect size; VE/VCO_2_, ventilatory equivalent of carbon dioxide.

### Early clinical outcomes (descriptive only)

Patients underwent open revascularisation in 23% of cases (n=14), endovascular intervention in 48% (n=29) and hybrid revascularisation in 20% (n=12). Primary major limb amputation was performed in 8% of cases (n=5).

Although not a primary outcome of this feasibility study, 30-day and 90-day clinical outcomes were recorded descriptively to characterise cohort risk. At 30 days, hospital readmission occurred in 17% (n=10), comparable to national data (14.8% open; 19.7% endovascular),^2^ with no all-cause mortality, lower than national data of 3.5–4.2% following revascularisation and 5.9% after major amputation.^2^ MALE occurred in 17% (n=10) and MACE in 3% (n=2). By 90 days, hospital readmission occurred in 57% (n=34), all-cause mortality was 2% (n=1), MALE 25% (n=15) and MACE 3% (n=2).

## Discussion

This study assessed the safety and feasibility of CPETarm in patients admitted non-electively with CLTI. The results demonstrate that CPET is feasible with minimal disruption to clinical pathways and is widely accepted by patients. These findings highlight its potential use as a preoperative risk stratification tool in this high-risk surgical cohort. Furthermore, these clinical outcome rates reinforce the high-risk profile of this patient cohort and support the need for objective perioperative risk stratification tools.

A recent multinational Delphi consensus study highlighted uncertainty regarding the optimal approach to individual risk assessment in CLTI,^35^ noting limitations of existing tools such as the American College of Surgeons National Surgical Quality Improvement Programme Surgical Risk Calculator and the Surgical Outcome Risk Tool, and lack of consensus on cardiac stress testing. In this context, CPETarm offers a novel, objective method to assess physiological reserve. This could facilitate more accurate risk stratification, allow for personalised perioperative planning, or, when necessary, a conservative management strategy supported by objective data.

Importantly, 90% of tests were completed within 48 hours of treatment decisions and testing did not delay access to intervention, a critical requirement in the management of patients with CLTI.^3^ Same-day referral, dedicated equipment and a standardised protocol facilitated rapid access. There was minimal disruption to clinical pathways, as testing was integrated alongside other assessments or while awaiting theatre availability. These findings highlight the practicality of CPETarm as a point-of-care assessment that can be integrated into the workflow without adversely impacting patient outcomes. Moreover, the test was well tolerated, and no serious adverse events were reported, reaffirming its safety profile even among patients with significant comorbidity, frailty and functional limitations.

Patient feedback supported the acceptability of CPETarm, with a preference over conventional forms of exercise testing such as a treadmill or ‘Timed Up and Go’ test. This is particularly relevant for patients with CLTI, who are frequently unable to mobilise. This is critical as patient willingness influences the success of implementation and applicability to real-world settings.

The study achieved a 95% completion rate, with 45% achieving an RER >1.15 and 35% attaining >80% of their age-predicted maximal heart rate. The relatively low proportion meeting heart rate-based criteria likely reflects the high prevalence of β-blocker therapy and the chronotropic incompetence characteristic of this cohort.^36^ There is no gold-standard definition for maximal effort during arm-ergometry CPET, and considerable variability exists in the significance assigned to commonly used objective markers.^6 22 26 37^ Accordingly, we reported multiple physiological thresholds to enhance transparency. Given the feasibility focus of the present study, effort criteria were not used as inclusion/exclusion for this analysis. In this context, the absolute values are less important than the overarching observation: patients historically excluded from objective physiological testing can safely exercise and generate physiologically plausible data, providing supportive, though not definitive, evidence of near-maximal exertion.

For patients unable to perform lower-limb exercise testing, arm ergometry may serve as a standalone modality. Our data demonstrates that CPETarm produced appropriate 9-panel plots supporting its physiological validity. Since arm-ergometry normative data are limited and derived largely from healthy, younger volunteers, direct application to the acute, multimorbid CLTI population is inappropriate.^38^ Larger studies are needed to establish normative values and prognostic thresholds in vascular inpatients rather than scaling from leg-based standards or from healthy volunteers.

Limitations of this study include its single-centre design and potential interviewer bias, given that patient feedback was collected by the CPET practitioner. Potential biases were mitigated through standardised procedures, transparent reporting of multiple thresholds and an independent review of audio recordings and transcripts by a second researcher independent from testing. The generalisability of our findings may be influenced by our specific service configuration. Our CPET provision is embedded within a preoperative assessment pathway, with same-day access for patients requiring urgent surgery, delivered in a dedicated testing space with specialised equipment and staff experienced in CPET interpretation. Centres without similar infrastructure, staffing expertise and integrated preoperative pathways may require additional resources, training and service design to deliver a comparable model. We also acknowledge that the sample size may limit generalisability and may not reflect variability in performance or wider patient responses to acceptability of CPETarm. In addition, there is a risk that the findings reflect a self-selected cohort of patients who were considered medically stable to undergo testing and provide consent for study enrolment. This may partly explain the more favourable outcomes observed at 30 and 90 days, although this study is too small to make direct comparisons to national data. Conversely, patients excluded from CPET are often those at highest perioperative risk and may therefore be less likely to benefit from further risk stratification. While inclusion of patient perspectives enhances the relevance and patient centredness of the study, further structured qualitative research from other members of the multidisciplinary team is needed to better understand barriers and preferences that may impact broader adoptions.

## Conclusion

In conclusion, this study has shown that CPET using arm ergometry is feasible, acceptable and safe in patients with CLTI as a point of care test. These results support further investigation into its clinical utility as a preoperative risk stratification tool, with particular focus on defining modality-specific prognostic thresholds. If validated, CPETarm could represent a significant advancement in the preoperative assessment of this high-risk surgical cohort, who are often excluded from traditional exercise testing modalities. This could improve shared decision-making, resource utilisation and may contribute to improved outcomes in this population.

## Data Availability

Data are available upon reasonable request.
