# Supplementary material for: Novel use of arm ergometry cardiopulmonary exercise testing for non-elective patients with chronic limb-threatening ischaemia: a feasibility trial
Source: BMJ Open. 2026 Jun 15;16(6):e118841. doi: 10.1136/bmjopen-2026-118841 (PMC13289131; doi:10.1136/bmjopen-2026-118841)
Supplement: Supplementary file 1 [file bmjopen-2026-118841.reviewer_comments.pdf]

PEER REVIEW HISTORY

BMJ Open publishes all reviews undertaken for accepted manuscripts. Reviewers are asked to complete a checklist review form and are provided with free text boxes to elaborate on their assessment. These free text comments are reproduced below.

ARTICLE DETAILS

Title (Provisional)

Novel use of arm ergometry Cardiopulmonary Exercise Testing for nonelective patients with chronic limb-threatening ischaemia: a feasibility trial

Authors

Hodge, Stacie; Haque, Adam; Quraishi-Akhtar, Tanviha; Bryan, Angella; Rogers, Steven; Bowling, Frank Lee; Ghosh, Jonathan

---

VERSION 1 - REVIEW

**Reviewer**	**1**
**Name**	**Highton, Patrick**
**Affiliation**	**University of Leicester, Cardiovascular Sciences**
**Date**	**10-Mar-2026**
**COI**	**None**

A very well written and interesting study, with clear implications for clinical benefit in this population. Just a few comments below.

Specific comments:

What is the value of statistically comparing baseline data between males and females – surely differences would be expected? BMI, grip strength etc.

Although the early clinical outcomes are descriptive only, can the authors comment on how this compares generally to this population after surgery?

General comments:

The limitation mentioned on page 4 regarding the single centre design and generalisability to other clinical settings seems key. Could the authors add more information to the discussion regarding how their clinic set up and expertise differs from a 'typical' clinic, and what sort of extra resources or training would need to be in place for other clinics to conduct this sort of testing?

**Reviewer**	**2**
**Name**	**Sadeghipour, Parham**
**Affiliation**	**Rajaie Cardiovascular Medical and Research Center, Vascular Disease and Thrombosis Research Center**
**Date**	**14-Mar-2026**
**COI**	**None**

Preoperative assessment for patients with CLTI remains controversial, with no specific or practical recommendations currently available. This represents an important knowledge gap, given the substantial cardiovascular burden among patients with CLTI and the need for careful perioperative risk evaluation. The authors should be congratulated for their effort to introduce a CPET-based approach for preoperative risk assessment.

My only minor comment is that the authors should provide information regarding the percentage of patients who ultimately underwent surgical or hybrid revascularization following CPET evaluation. In addition, it would be valuable to discuss potential threshold values that could help clinicians risk-stratify patients in clinical practice.

**Reviewer**	**3**
**Name**	**Brittenden, Julie**
**Affiliation**	**University of Glasgow, Institute of Cardiovascular Research**
**Date**	**23-Mar-2026**
**COI**	**None**

The authors have demonstrated that in well conducted feasibility study that arm ergometry cardiopulmonary exercise testing in patients with CLI is safe, practical and acceptable. It a single centre it was implemented effectively within an appropriate time frame.

I have a few queries:

1) Methodology

i) Could the authors provide a link please to the novel protocol used in this feasibility study please?

ii) How reproducible is arm ergometry in this cohort?

iii) It appears that the participants required to warm up by cycling rather than by arm exercises, is this correct? If so how did this impact on the bed side component of the test and willingness of patient with lower limb rest pain or ulceration to take part?

2) Inclusion & Exclusion criteria

I note the patients as anticipated from this cohort had significant comorbidity and 28% (17) had a clinical frailty score of 5 or more. Do the authors anticipate that the result of the arm ergometry will have an additive effect on frailty score in determining prognosis and would this be of value across categories or should those of high scores be excluded?

3) Were the results made available to the clinical team and did this impact on decision to operate or procedure undertaken. Did exclusion from CPET influence the management of these patients?

4) The authors acknowledge that further research is required to evaluate potential prognostic value and thresholds. What sort of patient numbers would need to be involved in such an evaluation?

VERSION 1 - AUTHOR RESPONSE

Reviewer: 1

1. What is the value of statistically comparing baseline data between males and females – surely differences would be expected? BMI, grip strength etc.

We have removed the text from the results section of the manuscript however we feel it is important to leave this comparison in tabular form.

Statistically comparing baseline characteristics between males and females remains important, even where differences might be anticipated. Rather than relying on assumptions, formal comparison allows us to quantify the magnitude of these differences and assess their potential clinical relevance within the specific study population. This is particularly pertinent for variables such as BMI, Haemoglobin and grip strength, where the degree of difference may directly influence protocol design or interpretation of CPET variables and outcome measures.

In our study, for example, identifying sex-related differences in grip strength informed optimisation of the CPET protocol and our interpretation of reference values, ensuring it was appropriately tailored rather than implicitly standardised around male-derived norms. Furthermore, there is a strong rationale for explicitly reporting these comparisons given the historical underrepresentation of women in clinical research, particularly in vascular surgery. Presenting sex-disaggregated baseline data helps address this gap and supports more generalisable and equitable conclusions.

2. Although the early clinical outcomes are descriptive only, can the authors comment on how this compares generally to this population after surgery?

We have now added contextual comparison with published data (lines 560-562 marked copy). The early clinical outcomes in our cohort are descriptive and the sample size in this feasibility study is too small to expect concordance with population data however the outcomes are broadly consistent with published data. At 30 days, our readmission rate of 17% aligns with national vascular registry data (14.8% for open and 19.7% for endovascular procedures), and the absence of 30-day mortality compares favourably to reported 3.6-4.2% for revascularisation and 5.9% following major amputation.

3. General comments:
The limitation mentioned on page 4 regarding the single centre design and generalisability to other clinical settings seems key. Could the authors add more information to the discussion regarding how their clinic set up and expertise differs from a 'typical' clinic, and what sort of extra resources or training would need to be in place for other clinics to conduct this sort of testing?

We now describe that our service was embedded within an established preoperative assessment pathway, with same-day access for urgent surgical patients, dedicated testing space and equipment, and staff experienced in CPET setup, testing and interpretation. We also note that centres without comparable infrastructure or expertise may require additional resources, training and service redesign to deliver a similar model (Lines 638-644marked copy). The CPET setup itself was relatively easy to set up, consisting of just an arm ergometer and a metabolic cart. Having dedicated in-house equipment enabled us to assess patients rapidly within the limited preoperative window before urgent surgery. The software configuration and testing protocol are reproducible in other centres, and we would be happy to make these available to support wider adoption.

Reviewer: 2

4. My only minor comment is that the authors should provide information regarding the percentage of patients who ultimately underwent surgical or hybrid revascularization following CPET evaluation. In addition, it would be valuable to discuss potential threshold values that could help clinicians risk-stratify patients in clinical practice.

Thank you for this suggestion. We have now included a detailed breakdown of procedures in the main body of the manuscript, including open, endovascular, hybrid and primary major amputation.

Regarding threshold values for clinical risk stratification, we agree that this is an important consideration. However, as this was a feasibility study, the sample size and design were not intended to define or validate prognostic cut-offs. This however is the overall aim of this research but currently there is insufficient sample to give thresholds with any degree of certainty. With this in mind, we would be hesitant to reference any threshold values based on our current data in case this is misinterpreted and applied clinically mistakenly.

<u>Reviewer: 3</u>

5. Methodology

i) Could the authors provide a link please to the novel protocol used in this feasibility study please?

The novel protocol was developed as part of this work by our study team. Protocols for arm ergometry that are published (Shakespeare J, Parkes E. Reference values for arm ergometry cardiopulmonary exercise testing (CPET) in healthy volunteers. BMJ Open Respir Res. 2025) were evidently unsuitable for this patient cohort when this feasibility work began, as most patients were unable to tolerate the increase in load beyond 1 minute.

In response, we developed a modified protocol tailored to the tolerability and functional limitations of this cohort. As this protocol represents a novel methodological contribution, we are currently preparing a separate manuscript describing its development and validation in detail.

ii) How reproducible is arm ergometry in this cohort?

Within the scope of this feasibility study, reproducibility was not formally assessed. The study population consisted of non-electively admitted patients requiring urgent surgical intervention, which precluded repeat testing at a second time point (e.g. 24–72 hours apart).

However, we sought to minimise variability by standardising key aspects of the CPET protocol, including the ergometer type, testing location, equipment calibration procedures, operator instructions, and the level of participant encouragement.

Future studies in more stable populations, such as elective patients with CLTI or intermittent claudication, would allow formal assessment of test–retest reproducibility of arm ergometry in this context.

iii) It appears that the participants required to warm up by cycling rather than by arm exercises, is this correct? If so how did this impact on the bed side component of the test and willingness of patient with lower limb rest pain or ulceration to take part?

The warmup was done on the arm ergometer. We have changed the wording to improve clarity. Thank you for highlighting this.

6. Inclusion & Exclusion criteria
I note the patients as anticipated from this cohort had significant comorbidity and 28% (17) had a clinical frailty score of 5 or more. Do the authors anticipate that the result of the arm ergometry will have an additive effect on frailty score in determining prognosis and would this be of value across categories or should those of high scores be excluded?

We know from existing evidence that patients with a Clinical Frailty Score >5 experience poorer outcomes—higher mortality, greater risk of amputation, and a higher likelihood of being deemed unfit for surgery. What we have seen as part of a larger body of work outside the scope of this feasibility study is that these high CFS patients also have significantly worse physiological performance in CPET variables like peak $V0_2$, maximum watts and oxygen pulse. Even small graduations in frailty from CFS 3 to 4 revealed large differences in physiological reserve. We hope that by combining current objective measures such as blood results, hand grip strength and CFS with CPET variables we will gain a more complete picture of physiological reserve allowing us to stratify risk more accurately, tailor peri operative planning and when necessary, justify a conservative approach with objective data.

CPET also provides diagnostic information that a CFS score cannot. For example, it can help identify underlying myocardial ischaemia, obstructive respiratory disease, and metabolic conditions.

7. Were the results made available to the clinical team and did this impact on decision to operate or procedure undertaken. Did exclusion from CPET influence the management of these patients?

The clinical team was not informed of participants' CPET results and remained independent of the research trial, therefore, CPET findings did not influence surgical decision-making or perioperative management. I have now included this in the methods section to improve clarity (lines 268-269 marked copy. Clinical adverse events were however disclosed ot the clinical team if identified. This only occurred in 2 patients as described in lines 447-459 marked copy.

8. The authors acknowledge that further research is required to evaluate potential prognostic value and thresholds. What sort of patient numbers would need to be involved in such an evaluation?

There is no fixed sample size for such evaluations, rather, predictive models generally improve in accuracy as the number of included patients increases, with iterative refinement over time. Drawing on our centre's related work in CPET risk stratification for abdominal aortic aneurysm surgery initial modelling was based on a cohort of 415 patients (Preoperative cardiopulmonary exercise testing and risk of early mortality following abdominal aortic aneurysm repair by R. A. Hartley et al., *Br J Surg*, 2012). This suggests that a cohort of a similar size would be sufficient for developing the initial model.

The next phase of our work will involve collaboration between at least two major vascular centres in the UK. Based on current recruitment rates, achieving a cohort of this size is feasible within approximately 2 years.

VERSION 2 - REVIEW

**Reviewer**	**1**
**Name**	**Highton, Patrick**
**Affiliation**	**University of Leicester, Cardiovascular Sciences**
**Date**	**30-Apr-2026**
**COI**	

All comments addressed

Reviewer **2**

Name **Sadeghipour, Parham**

Affiliation **Rajaie Cardiovascular Medical and Research Center, Vascular Disease and Thrombosis Research Center**

Date **30-Apr-2026**

COI

The authors responded to my previous comments, thank you

Reviewer **3**

Name **Brittenden, Julie**

Affiliation **University of Glasgow, Institute of Cardiovascular Research**

Date **14-May-2026**

COI

Thank you for comprehensibly addressing my queries. I am content with the changes and additional information provided
